# Multiphase Ozone Oxidation of Catechol and Its Products after OH- and Light-Driven Processing

**DOI:** 10.1021/acsearthspacechem.5c00230

**Published:** 2025-11-07

**Authors:** Sithumi M. Liyanage, Meredith Schervish, Habeeb H. Al-Mashala, Katrina L. Betz, Akansha Sharma, Manabu Shiraiwa, Elijah G. Schnitzler

**Affiliations:** a Department of Chemistry, 7618 Oklahoma State University, Stillwater, Oklahoma 74078, United States; b Department of Chemistry, 8788 University of California Irvine, Irvine, California 92697, United States

**Keywords:** reactive uptake, ozone, phenolic compounds, secondary organic aerosol, brown carbon

## Abstract

Phenolic compounds are some of the most abundant emissions of biomass burning during wildfires. Catechol, the most abundant isomer of benzenediol in biomass burning emissions, undergoes oxidation in the aqueous phase of cloud droplets to form secondary organic aerosol (SOA), including products that absorb light at visible wavelengths, called brown carbon (BrC) chromophores. After cloud evaporation, the remaining submicron SOA particles are susceptible to further oxidant- and light-driven processing. Here, we investigate the multiphase ozone oxidation of the reaction mixture from the aqueous OH-initiated oxidation of catechol, i.e., simulated OH-driven processing in clouds, using a coated-wall flow-tube apparatus. Reactive uptake of ozone was determined for thin films from the OH-driven processing of catechol with and without further irradiation of the thin films, i.e., simulated light-driven processing after cloud evaporation, at low and moderate relative humidity (RH). The experimental time series were reproduced using kinetic multilayer modeling, which, along with qualitative microscopy experiments, provided insights into the diffusivity of these materials. After OH-driven processing, the thin films exhibited uptake coefficients of 2 × 10^–6^ and 9 × 10^–6^ at 0 and 50% RH, respectively, and 4 h of exposure to 130 ppb of ozone. After OH- and light-driven processing, the uptake coefficients were lower, 2 × 10^–7^ at 0% RH and 4 × 10^–6^ at 50% RH, for the same ozone exposure. Consequently, the reaction mixture of catechol was plasticized upon uptake of water vapor but vitrified under UV irradiation. Kinetic multilayer modeling shows that slower ozone diffusion at low RH and after light-driven processing can lead to an increase in the atmospheric lifetime of reactive species from less than 1 h to more than a day.

## Graphical abstract

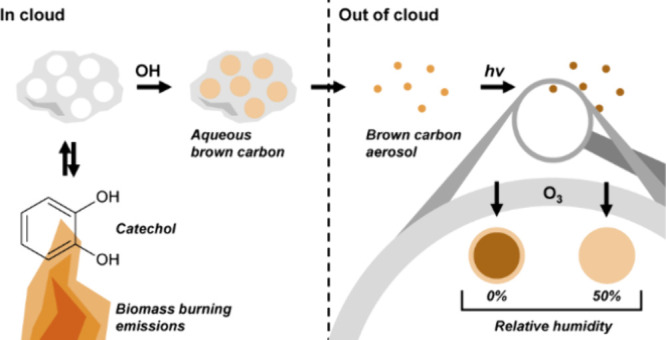

## Introduction

Phenolic compounds are abundant emissions of biomass burning, produced from the thermal degradation of lignin, a natural polymer of cross-linked phenolic units.^1–3^ Benzenediols are one of the most abundant classes of phenolic emissions of biomass burning.^1^ They are semivolatile, so they are distributed across the gas and particle phases, according to their saturation vapor pressures and, in turn, saturation concentrations.^1,4,5^ Catechol (i.e., 1,2-benzenediol) is the most abundant of the three structural isomers among the benzenediols in the emissions of burning pine, oak, and eucalyptus.^1^ It is also the most volatile, residing mostly in the gas phase,^1^ where it can undergo oxidation^6–9^ or readily partition into cloud droplets, according to its Henry’s law constant.^10–12^ Catechol secondary organic aerosol (SOA) is formed from oxidation initiated by ozone, hydroxyl (OH) radical, and nitrate (NO_3_) radical.^13–18^ Aqueous-phase cloud chemistry of catechol leading to SOA has also been studied for oxidation initiated by ozone, OH radical, and organic compounds in their triplet excited electronic states.^19–21^ Reaction of catechol with ozone at the air–water interface, representing the surface of cloud droplets, has also been studied.^22^ More generally, in-cloud processing of phenolic emissions of biomass burning has been observed in the field.^23–25^

Cloud droplet evaporation leaves behind submicron particles of SOA from the preceding aqueous-phase chemistry,^26–28^ which can lead to a wide range of products from catechol. For example, muconic acid is a well-known product of the ozonolysis of catechol.^29,30^ Smaller carboxylic acids are also known to form from oxidation of phenolic compounds.^31,32^ Other carbonyl-containing nonacid compounds, containing ketones, aldehydes, and quinones, have been found to be abundant products of the aqueous OH-initiated oxidation of phenolic precursors, as well.^33^ Larger molecules, including bi- and terphenyl and ether-linked aromatics, have been observed to form from individual phenolic compounds during oxidation, through oligomerization.^34–36^ Oligomers have also been observed to form from mixed components of biomass burning emissions, including phenolic compounds, during simulated cloud processing.^37^ The processes of functionalization and oligomerization result in products that absorb visible light more strongly than their phenolic precursors, i.e., secondary brown carbon (BrC) chromophores.^38,39^ After evaporation, these particulate species continue to age in the atmosphere through interactions with gas-phase oxidants, including ozone and OH radical, and sunlight. The multiphase processing of secondary BrC from phenolic precursors depends on the physical properties of the particles,^40^ e.g., viscosity governs the diffusion of oxidants from the surface into the particle bulk and organic components from the bulk to the surface. The viscosity of oxygenated organic compounds is known to depend on relative humidity (RH).^41,42^ Light-absorbing carbonyl-containing compounds, including quinones, can undergo Norrish reactions to produce carbon-centered radicals, which may drive further oligomerization and potentially alter the optical and physical properties of the particles.^43^

Here, we focus on the following hypotheses: first, the reaction mixture formed from aqueous OH-initiated oxidation of catechol and its products followed by solvent evaporation will exhibit reactive uptake of gas-phase ozone that varies significantly with RH; second, this reaction mixture will evolve significantly in terms of both absorptivity and reactive uptake of ozone upon subsequent light-driven processing, i.e., exposure of thin films to UV radiation after the initial preparation through OH oxidation. The reaction mixture, including secondary BrC chromophores, was generated in the laboratory by aqueous OH-initiated photo-oxidation of catechol in a photoreactor. The reaction mixture was isolated from solution by rotary evaporation of water and used to generate thin films in glass substrate tubes. Some samples were placed directly in a coated-wall flow-tube apparatus and analyzed to determine reactive uptake of ozone from the gas phase under controlled RH conditions. Other samples were exposed to further UV-B radiation as thin films before being analyzed in the coated-wall flow tube. Experimental time series of the uptake coefficient were simulated using kinetic multilayer modeling to determine diffusion coefficients within the thin films, and the lifetime of reactive species within atmospheric aerosol particles was simulated based on the resulting diffusion coefficients. This combination of experimental and computational approaches has recently been used for biomass burning organic aerosol (BBOA),^44^ but this is its first application to secondary BrC. Our results and their atmospheric implications are discussed below.

## Materials and Methods

### Aqueous OH Oxidation

Aqueous OH oxidation of catechol was performed in a custom-built photoreactor.^44–46^ The photoreactor consists of 16 UV-B bulbs (Ushio, G8T5E), with peak emission at 310 nm, arranged vertically along the inner wall of a cylindrical aluminum support that is 36 cm high and 25 cm in diameter, as shown in Figure S1. All 16 bulbs were powered throughout these experiments. Aqueous samples were placed in a 250 mL glass reagent bottle, along with a magnetic stir bar. The stir bar was spun at 400 rpm while the bottle was positioned at the center of the cylindrical photoreactor. The sample was protected from radiation with wavelengths shorter than 300 nm by the glass. Supported at the top of the photoreactor, a cooling fan (Noctua, NF-P14r) rotated at 1500 rpm; further cooling was provided by two large box fans positioned perpendicular to each other outside the aluminum enclosure. Blackout cloth (Thorlabs, BK5) was used to cover the box fans and enclosure. The temperature at the wall of the reagent bottle after the photoreactor equilibrated was 311 K.

Using ultrapure water (18.2 MΩ cm) from a commercial water purification system (Thermo, Smart2Pure 3 UV), 250 mL aqueous solutions of 4 mM catechol (Sigma-Aldrich, ≥99%) and 100 mM hydrogen peroxide (Fisher, 30% w/w) were prepared. These concentrations are higher than used in several previous studies of aqueous photo-oxidation of phenolic species,^31,46^ in order to generate sufficient material for the subsequent coated-wall flow-tube experiments. The solution of catechol and hydrogen peroxide was exposed to radiation in the photoreactor for 36 h. This length of time was chosen because it gave a significant change in the light absorption of the solution. Figure S2 depicts the solution before and after photo-oxidation. The reaction mixture that resulted from this period of photo-oxidation was then transferred without delay to a rotary evaporator (BUCHI, R-210), as shown in Figure S3, to remove the solvent.

### Thin-Film Preparation

After the solvent was removed from the reaction mixture in the rotary evaporator, the remaining material was used to prepare thin films for measurements of the reactive uptake of ozone. The reaction mixture residue was transferred to a vial by dissolving in methanol (Fisher, >99.8%), passed through a 0.45 μm syringe filter, and blown down with nitrogen (Airgas, Ultra High Purity) at 3 L min^–1^ to determine the mass of the residue gravimetrically, using an analytical balance. After weighing, the reaction mixture was reconstituted in isopropanol (Fisher, >99.5%) to give a solution with a mass concentration of 10 mg mL^–1^. A 4 mL aliquot of this solution was then dispensed in a glass substrate tube, between two machined and sealed Teflon spacers, and the glass tube was placed on a level roller mixer (Olde Midway, CON-ROLL-PRO18) that rotated slowly at room temperature. As a result, the glass tube contained 40 mg of the reaction mixture. Clean, filtered air was passed through the glass tube to enhance the evaporation of isopropanol. The thin films resulting from this procedure were uniform, transparent, and colored, as shown in Figure S4. The volume of organic material in the thin films was estimated from the mass and an assumed density of 1.2 g cm^–3^. The length of the prepared films was 7 cm, i.e., the separation between the sealed spacers. The thickness of the thin films was computed by dividing the estimated volume of organic material by the surface area coated. Between preparation and use, thin film samples were stored under nitrogen (Airgas, Ultra High Purity) in a stainless-steel tube, i.e., in the absence of light and oxidants.

### UV Irradiation

Some samples were further processed after preparation as thin films by exposure to UV irradiation for 16 h. This length of time was chosen because it was previously optimized to give significant changes in color for other samples in thin films, i.e., primary BrC.^44,45^ The glass tube was positioned vertically at the radial center of the cylindrical photoreactor, described above. Previously, chemical actinometry was performed for the reactor to show that this interval of irradiation is equivalent to 3 days (i.e., including day–night cycles by comparison to 24 h averaged, rather than noon, solar spectral flux) in the atmosphere.^45^ A representative thin film after exposure to UV irradiation is shown in Figure S4.

### Reactive Uptake Measurement

A coated-wall flow tube, illustrated in Figure 1, was used to quantify the reactive uptake of ozone to the prepared thin films. Ozone was produced using a Hg lamp (UVP, SOG-2) and first found to be stable at 130 ppb using a UV absorption analyzer (Ecotech, Serinus 10). A temperature and RH probe (Vaisala, HMP75) was used to monitor the RH, which was adjusted to either 0 or 50%. Experiments were conducted at low and moderate RH because at higher RH, the thin films deliquesced and coalesced into large droplets, with unknown surface area. Air from a zero-air generator (Aadco, 747-30) was passed through the ozone generator, a humification setup, and a retractable stainless-steel injector, which was precisely centered, at 0.3 L min^–1^. The total flow rate was set by a mass flow controller (Sensirion, SFM5500 Series), and the flow rates through the respective ozone generation and humidification branches of the setup were set by needle valves on rotameters. All flow rates were verified using a bubble-based calibrator (Sensidyne, Gilibrator 2). After passing through the temperature and RH probe, air leaving the flow tube was sampled by the ozone analyzer. The analyzer sampled at a flow rate of 0.4 L min^–1^, so the remaining 0.1 L min^–1^ of the overall flow rate was supplied from the lab through a tube packed with ozone-destroying catalyst (Carus, Carulite 200). The mixing ratio of ozone was recorded in LabVIEW.

**Figure 1:**
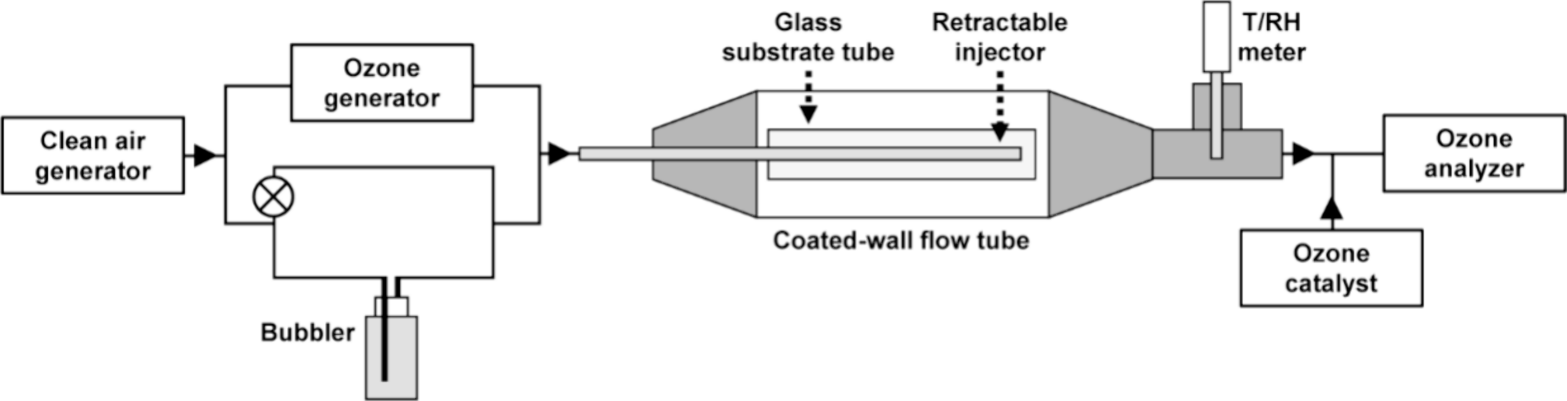
Schematic of experimental setup for the coated-wall flow tube.

Before inserting the coated glass substrate tube into the apparatus, the injector was fully retracted. Clean air, with no introduction of ozone or water vapor, had been flowing through the apparatus overnight beforehand. After inserting the substrate tube, the thin film was conditioned for 30 min at 0% RH to prepare for ozone exposure at 0% RH. After conditioning, the injector was translated forward such that it was aligned with the far edge of the film, and the ozone generation began, until a mixing ratio of 130 ppb was achieved. Once the ozone mixing ratio was steady, the injector was retracted 5 cm over the thin film. After 4 h, the injector was returned to its initial position. To allow for fitting, in the event of slight baseline drift between the beginning and end of the experiment,^47,48^ the ozone mixing ratio was recorded for at least 30 min after the injector was moved forward. The same procedure was followed for ozone exposure at 50% RH. Table S1 lists a series of parameters for the setup.

After an experiment, the ratio of the real-time ozone mixing ratio, [O_3_], to the initial mixing ratio, [O_3_]_0_, was used to calculate the first-order rate constant of reactive uptake, *k*_obs_.

$$k_{\mathrm{obs}}=-\frac{\ln\left(\frac{\left[O_{3}\right]}{{\left[O_{3}\right]}_{0}}\right)}{t}$$

Here, *t* is the residence time of ozone over the thin film. The effective uptake coefficient, γ_eff_, which is the ratio of reactive uptake to total collisions of ozone with the surface, was determined from *k*_obs_, as well as the diameter of the coated glass tube, *D*_tube_, and the mean speed of ozone, ω_ozone_, at the experimental temperature.

$$\gamma_{\mathrm{eff}}=\frac{D_{\mathrm{tube}}k_{\mathrm{obs}}}{\omega_{}}$$

Finally, the corrected true uptake coefficient, γ_corr_, was calculated from γ_eff_ as follows:^49^

$$\gamma_{\mathrm{corr}}=\frac{\gamma_{\mathrm{eff}}}{1-\gamma_{\mathrm{eff}}\frac{3}{2N_{\mathrm{Shw}}^{\mathrm{eff}}\mathrm{Kn}}}$$

Here, *N*_Shw_^eff^ is the effective Sherwood number, accounting for mass-transfer limitations arising from diffusion of ozone in the gas phase, and Kn is the Knudsen number, accounting for flow regime.

### Spectroscopic and Chemical Analyses

UV–vis absorbance was measured for the reaction mixture in aqueous solutions before and after initial photo-oxidation and in isopropanol solutions before and after irradiation of the thin films, using a modular fiber-optic-based spectrometer. Dilutions were performed as required and accounted for precisely, so all mass concentrations were known. The spectrometer consisted of a balanced deuterium-halogen light source (DH-2000-BAL), a 1 cm cuvette holder and lenses (Thorlabs, CVH100), and a miniature spectrometer (Ocean Insight, Flame-T-UV–vis). Before spectra were collected, the light source was given at least 1 h to warm up and stabilize. Minutes before each sample spectrum was recorded, using Ocean ART software, a reference spectrum was obtained.

In select experiments, the concentration of catechol during aqueous OH-initiated oxidation in the photoreactor was monitored using liquid chromatography (Agilent, 1260 Infinity), with a 1.8 μm C18 column (Agilent, Extend-C18, 2.1 × 50 mm), coupled to a diode array detector (Agilent, 1260 DAD HS). The column temperature was set to 298 K. A gradient elution was used at a flow rate of 0.3 mL min^–1^: at 0 min, the mobile phase was 90% ultrapure water and 10% methanol (Fisher Optima, ≥99.9%); over 20 min, the contribution of methanol was increased linearly to 100% and held constant for 5 min; finally, over 2 min, the contribution of methanol was returned linearly to 10% and held constant for 8 min. The diode array detector was balanced immediately before each sample, and it recorded absorbance across the range of 190 to 640 nm. Quantification was performed at 280 nm, where catechol exhibits strong UV absorption, with a bandwidth of 10 nm.

### Microscopy

Microscopy was used to qualitatively probe viscosity, using a method described previously.^44^ For these observations, thin films were prepared on flat circular glass coverslips (VWR, 48382-042), rather than the glass substrate tubes used for the uptake experiments, with the same thickness. After an aliquot of the reaction mixture from the substrate tubes dissolved in isopropanol, with a mass concentration of 10 mg mL^–1^, was dispensed onto a coverslip, the isopropanol was left to evaporate. Samples for microscopy were prepared from the reaction mixture in fresh and irradiated thin films extracted from the glass substrate tubes. The thin films were examined using a trinocular compound microscope (AmScope, CLT490B) at 298 K and 35% RH. The qualitative microscopy experiments began by scraping the thin film with the square edge of the end of a precision cutting blade. Micrographs were recorded 0, 20, and 120 min after scraping the film, so the appearance of the scrape could be monitored.

### Kinetic Multilayer Modeling

The kinetic multilayer model of gas-particle interactions in aerosols and clouds (KM-GAP)^50^ was used to simulate the flow tube conditions and to reproduce the measured uptake coefficients of ozone to the thin films of OH-initiated photo-oxidized catechol with and without additional irradiation under different RH conditions. KM-GAP was then used to simulate the lifetime of reactive components in atmospheric aerosol particles exposed to atmospherically relevant ozone mixing ratios. The details of KM-GAP and of its application to flow tube experiments have been described previously,^50–52^ but a brief description is given here as well as the specifics of this implementation.

KM-GAP consists of a gas phase, a near-surface gas phase, a sorption layer, a near-surface bulk layer, and a number of bulk layers. KM-GAP explicitly treats the processes of gas-phase diffusion, adsorption, and desorption, surface-to-bulk transport, and bulk diffusion within the thin film or particle. The interfacial reaction between ozone and reactive components in the thin film is simulated in the near-surface bulk layer, and bulk reactions are treated in all the bulk layers of the film and aerosol particles. In consistency with previous studies,^44,53^ we did not explicitly treat reactions of adsorbed ozone to reduce complexity of the model; inclusion of a surface reaction would likely enhance uptake and thus necessitate lower simulated bulk diffusivities to achieve good agreement. Therefore, the bulk diffusivities presented here likely represent upper bounds in this regard. The reaction of the model reactive component and ozone to give the product is the only chemical transformation treated. The reaction product was assumed to be nonvolatile and have a molecular weight of 248 g mol^–1^ and density of 1.3 g cm^–3^;^54,55^ the value 248 g mol^–1^ is the median of the molecular weights for BrC components from pine combustion, which ranged from 138 to 358 g mol^–1^,^54^ as rationalized earlier.^53,56^ The Henry’s law constant for ozone in the biomass burning material film was set at 2.4 × 10^–4^ mol cm^–3^ atm^–1^, as in previous work.^44,53^ This value is chosen based on experimental data to be between the Henry’s law constant of ozone in oleic acid and water, as a value for catechol secondary organic material is unknown. We conducted a sensitivity study with a factor of 2 higher Henry’s law constant for ozone under low RH without further irradiation, as ozone, being hydrophobic, may be more soluble in films with lower water content.^57^

For the simulated flow tube setup, the number of layers in the film bulk was set to 100 and a flat geometry was implemented by using a constant surface area for each layer. As in the experimental setup, the film was exposed to 130 ppb of ozone, which was constantly replenished at the experimental flow rate. The ozone uptake coefficient was calculated as the difference between the adsorption and desorption flux divided by the collision flux.^50^ The second-order rate coefficient for the reaction of ozone with the reactive organic species in the film was fixed to 5 × 10^–17^ cm^3^ s^–1^, and the number concentration of reactive components in the film was fixed to 3 × 10^16^ cm^–3^ as in previous work.^44^ The simulated fit was sensitive to total reactivity, and therefore, the rate constant and initial concentration of reactive components could not be constrained independently. A sensitivity study was performed investigating an order of magnitude lower initial concentration of reactive components in the irradiated films as light-initiated reactions may lead to lower subsequent reactivity toward ozone. The ozone and reactive component bulk diffusivities in the film were determined from the best fit to the experimental uptake coefficient data assuming the Henry’s law constant, second-order reaction rate coefficient, and initial concentration of reactive components given above. It is important to note that the simulated bulk diffusivities are highly dependent on these fixed parameters and thus cannot be fully constrained without more specific knowledge of these values. For example, a factor-of-two increase in the Henry’s law constant for ozone results in a roughly factor-of-two decrease in the best fit diffusion coefficient for ozone for all cases. Therefore, while the values of the bulk diffusivities from the best fit will be presented, the discussion in this work will focus on the relative order of the bulk diffusivities under different conditions.

To calculate BrC lifetimes in atmospheric aerosol particles, particles with diameter of 300 nm were exposed to 35 ppb of ozone. The diameter was chosen to be representative of SOA particles from in-cloud processing; e.g., for dried SOA particles formed during a cloud event in a chamber, a mode centered at 300 nm was observed.^28^ The number of layers in the particle bulk was set to be 10. The best-fit values for the diffusivities of ozone and the reactive species in the catechol secondary organic material in each experiment were used to simulate the decay of reactive species due to reaction with ozone in the particle. The lifetime of the reactive species was calculated as the *e*-folding time scale.

## Results and Discussion

### Effects of Relative Humidity

Upon aqueous OH-initiated photo-oxidation, secondary BrC formed from the initially colorless solutions of catechol. The formation of oxidation products that absorb light at visible wavelengths is evident qualitatively in Figure S2 and quantitatively, in terms of absorbance, in Figure S5. After 36 h of photo-oxidation, on average, about 25% of the initial catechol remained in solution, as determined by liquid chromatography. The formation of SOA from catechol, among other phenol and methoxyphenol emissions of biomass burning,^1^ has been studied extensively in the past, as described above, both in the gas and aqueous phases.^13,14,20^ The initial increase in absorptivity as secondary BrC forms is well known to occur due to functionalization and oligomerization.^32,35,58^ For example, the aqueous and multiphase OH-initiated photo-oxidation of catechol has been shown to result in the formation of oxygenated biphenyl and terphenyl compounds,^22,59,60^ which exhibit more light absorption than the constituent phenolic monomers through delocalization across the aromatic rings.^61^ After cloud droplet evaporation, these polar species are expected to readily absorb water from the gas phase, so the particles may be plasticized by elevated RH.^62,63^

We first investigated the effects of RH on the reactive uptake of ozone to the reaction mixture for thin films without further irradiation on the glass substrate tubes. At 0% RH, the ozone mixing ratio initially decreased to about 0.65 of the background mixing ratio, as shown in Figure 2a. The relative mixing ratio began increasing, eventually approaching an asymptote of about 0.9 by the end of the 4 h exposure experiment. As shown in Figure 3a, the initial mixing ratio corresponds to an uptake coefficient (including all corrections described above) of about 8 × 10^–6^; i.e., eight out of every one million collisions lead to reaction and loss of an ozone molecule. At the end of the 4 h exposure, the uptake coefficient reached a roughly stable value of 2 × 10^–6^. The reactive uptake is also shown in a log–log plot in Figure S6; beginning at about 1 h, the slope is −0.66, similar to the value (−0.5) that is characteristic of bulk diffusion-limited uptake.^52^ This uptake at 0% RH to the reaction mixture, which includes secondary BrC, was less than previously observed for primary BrC, i.e., BBOA from eastern red cedar. For whole BBOA, including compounds across a wide range of polarities (i.e., soluble in methanol), the ozone mixing ratio dropped to about 0.5, for the same operating conditions and geometry, yielding an uptake coefficient of nearly 1.5 × 10^–5^.^45^ For water-soluble and -insoluble components of BBOA at 0% RH, the relative ozone mixing ratio initially reached about 0.60 and 0.65, respectively, corresponding to uptake coefficients both on the order of 10^–5^.^44^ The components of the reaction mixture here were all water-soluble, so we did not separate the mixture by polarity, as we previously did for BBOA.^44^

**Figure 2:**
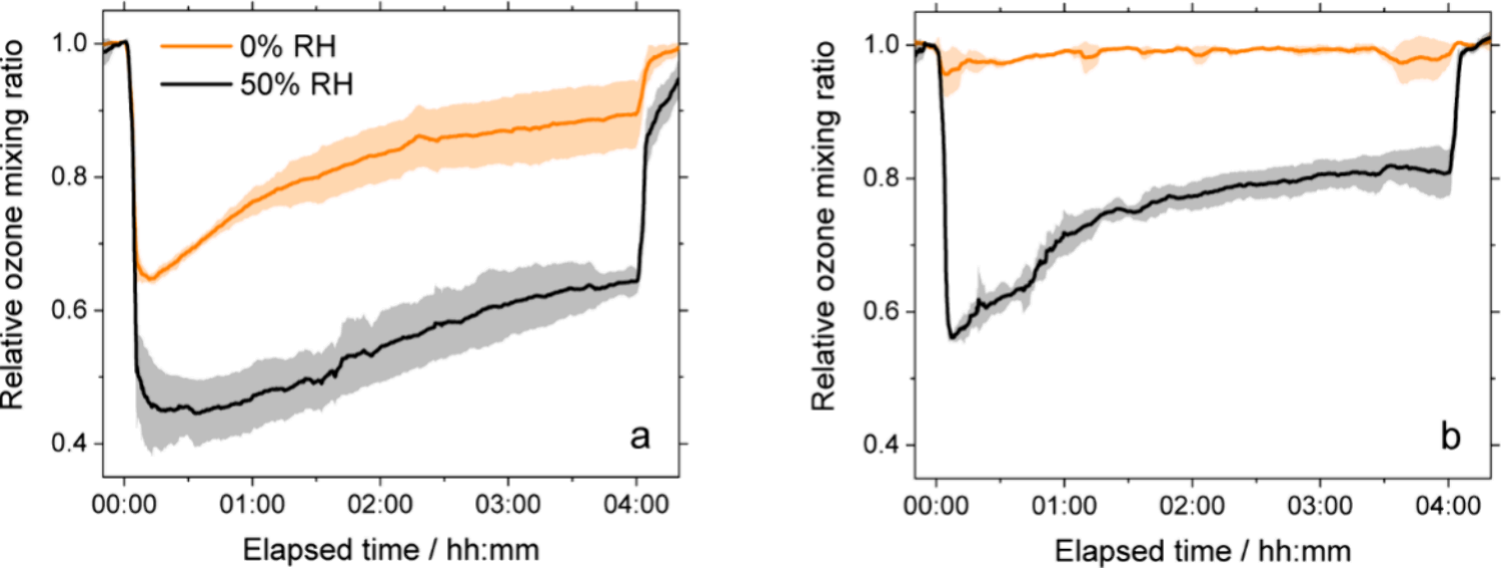
Relative ozone mixing ratios as a function of exposure time in the flow tube (a) without and (b) with further irradiation of the thin films. The shaded regions depict variance between triplicate experiments, in terms of one standard deviation.

**Figure 3:**
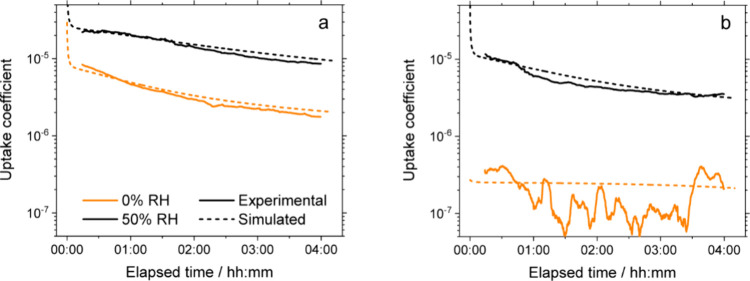
Uptake coefficients as a function of exposure time in the flow tube (a) without and (b) with further irradiation of the thin films. Each experimental time series is the average of triplicate experiments. The variance between triplicates is omitted here for clarity of presentation, but it is shown in terms of one standard deviation in Figure S7.

At 50% RH, the ozone mixing ratio initially decreased to 0.45 of the background mixing ratio, corresponding to a large increase in the uptake coefficient, which reached 2.3 × 10^–5^. Previously, we have observed reactive uptake to water-soluble and water-insoluble BBOA at 75% RH.^44,45^ As described above, we could not perform the reactive uptake experiments here for this reaction mixture at 75% RH, because the thin films deliquesced, an indication of their high hygroscopicity, and resulted in large droplets with an unknown surface area. Although the elevated RH used here is not as high as 75% RH, the observed relative mixing ratio is still between those observed for water-soluble and water-insoluble BBOA, 0.4 and 0.5, respectively.^44^ These earlier values corresponded to uptake coefficients of about 3 × 10^–5^ and 2 × 10^–5^, respectively.^44^ The significant increase in reactive uptake from 0 to 50% RH is consistent with the components of the reaction mixture being highly water-soluble. In contrast, the reactive uptake of water-insoluble components of BBOA was largely unaffected by increases from 0 to 75% RH, attributed to weak interactions with water.^44^ After 4 h of exposure, the relative mixing ratio of ozone increased to about 0.65 (see Figure 2a), and the uptake coefficient decreased significantly to about 9 × 10^–6^ (see Figure 3a).

The reactive uptake of ozone in the experiments was simulated using kinetic multilayer modeling to estimate values of the diffusion coefficients governing the multiphase processing.^53^ These values and others are listed in Table S2. At 0% RH, the diffusion coefficient of ozone in the thin film was found to 4.5 × 10^–8^ cm^2^ s^–1^. At 50% RH, this value increased by an order of magnitude, up to 4.5 × 10^–7^ cm^2^ s^–1^. The diffusion coefficient of the reactive components of the thin film, including secondary BrC chromophores, could lie in the range of 1 × 10^–20^ to 1 × 10^–10^ cm^2^ s^–1^, as values across this range did not impact the agreement between the experimental and simulated time series. Since ozone has a relatively low Henry’s law constant in water,^64^ the solubility of ozone in the reaction mixture may be higher at lower RH,^57^ so we also explore the effect of increasing the Henry’s law constant of ozone on the simulated reactive uptake and resulting ozone diffusion coefficients. As a result of increasing the Henry’s law constant by a factor of 2, to give 4.8 × 10^–4^ mol cm^–3^ atm^–1^, the ozone diffusion coefficient at 0% RH for the thin films without further irradiation decreased from 4.5 × 10^–8^ to 2 × 10^–8^ cm^2^ s^–1^ (see Table S3), indicating that a higher solubility of ozone at low RH would require lower diffusivity to give similar simulated time series (see Figure S8). Consequently, for this material (and the irradiated thin films, discussed below), while the assumption of a fixed Henry’s law constant at different RHs does affect the absolute value of the simulated bulk diffusivity that leads to agreement with the data, it does not influence the relative order of the magnitudes of ozone diffusion coefficients, which decrease with RH.

The value of the ozone diffusion coefficient at 0% RH found here is on the same order as the values observed for water-soluble (1.8 × 10^–8^ cm^2^ s^–1^) and water-insoluble (2.8 × 10^–8^ cm^2^ s^–1^) BBOA, while the wide range of the diffusion coefficient of the reactive organics, 10^–20^–10^–10^ cm^2^ s^–1^, extends below the range observed for BBOA: 1 × 10^–10^ cm^2^ s^–1^ for water-soluble and 8 × 10^–10^ cm^2^ s^–1^ for water-insoluble components.^44^ In other words, it may be more difficult here for unreacted organic components in the bulk layers to diffuse through the thin film to replenish the surface and near-surface layers. A diffusion coefficient of the reactive organics that is lower for secondary than primary BrC is consistent with past observations.^40,56,65^ For instance, the viscosity (i.e., inversely proportional to diffusion coefficient) of SOA from OH-initiated oxidation of catechol in the gas phase was determined to be about four orders of magnitude higher than that of BBOA from the thermal degradation of pine.^56,65^ Furthermore, the range found here, regardless of RH, encompasses the values found in smog chamber experiments for secondary BrC aerosol from the OH-initiated oxidation of resorcinol (i.e., an isomer of catechol) in the aqueous phase; these earlier values were 1 × 10^–16^ cm^2^ s^–1^ at 0% RH and 1 × 10^–14^ cm^2^ s^–1^ at 60% RH.^40^

### Effects of Irradiation

Upon further irradiation of the thin films, the light absorption of the deposited reaction mixture increased. This absorption enhancement of the thin films is evident qualitatively in Figure S4 and quantitatively, in terms of absorbance, in Figure S9. Similar increases in absorptivity through light-driven processing have been observed for primary BrC, i.e., BBOA from pine and eastern red cedar,^45,66,67^ and they have implications for the climate forcing of these materials in the atmosphere.^68–70^ Carbonyl-containing compounds are abundant among the products of aqueous OH-initiated photo-oxidation of phenolic compounds,^33,35,36^ i.e., the material used here to prepare the thin films before further irradiation. Based on Fourier-transform infrared (FT-IR) spectroscopy, carbonyl-containing functional groups account for nearly 10% of the mass of SOA from aqueous OH-initiated oxidation of phenol (i.e., a precursor closely related to catechol), and only about 20% of this contribution is due to carboxylic acids.^33^ Carbonyls, including quinones, were also detected using FT-IR spectroscopy in SOA from gas-phase OH-initiated oxidation of specifically catechol.^14,71^ These compounds, including quinones, often absorb visible light and can undergo direct photochemistry through Norrish reactions to produce carbon-centered radicals.^72^ These radicals can react with the other carbonaceous components of the reaction mixture to form oligomers, which have been shown to absorb more light but may also increase the viscosity of the materials.^43,61^

We consequently explored the effects of this further irradiation on the multiphase ozone oxidation of the thin films prepared from the catechol reaction mixture. Irradiation of the thin films for 16 h in the photoreactor led to samples that exhibited very little reactive uptake of ozone at 0% RH, as shown in Figure 2b. In terms of the uptake coefficient, the values were less than 10^–6^ for the whole duration of the 4 h exposure, as shown in Figure 3b. This uptake is slightly lower than previously observed for water-insoluble components of BBOA under the same conditions but is comparable to the water-soluble components, which also exhibited uptake coefficients below 10^–6^ throughout the exposure.^44^ In principle, these low uptake coefficients could be explored more precisely by adjusting the experimental conditions (e.g., increasing the length of the thin film), but for the purposes of comparison between the four conditioning schemes, we used the same experimental parameters for all samples (see Table S1). In contrast, at 50% RH, appreciable uptake still occurred after further irradiation, but it was significantly reduced from the 50% RH experiment without irradiation of the thin film. The relative ozone mixing ratio dropped to about 0.6, initially. The initial drop corresponds to an uptake coefficient greater than 10^–5^, about half that observed for the thin film without further irradiation. The fast recovery, within just 1 h, to approach a mixing ratio of 0.8 corresponds to a steady-state uptake coefficient of less than 5 × 10^–6^.

The significant difference upon irradiation could be due to increased viscosity. We explored the viscosity of the thin films qualitatively using microscopy, as described previously.^44^ As shown in Figure S10, without irradiation, scraping the thin film at 35% RH immediately (i.e., at 0 min) resulted in a narrow channel with distinct but rounded edges. After 20 min, the channel became narrower, and the edges became more diffuse. After 120 min, the channel almost closed, just distinguishable from the rest of the thin film. After irradiation, scraping initially resulted in a jagged channel. Adhesion is evident from the tags of organic material that had recoiled to rest on top of the initial layer of the thin film. Material also adhered to the scraping blade, so the channel was considerable wider than that observed without irradiation (see Figure S10). After 20 min, some flow was observed, e.g., the tag in the bottom-left quadrant was barely visible, having settled into the underlying thin film. After 120 min, further flow was evident in that the edges were less sharp and, in the bottom-right quadrant, a second, larger tag had almost settled into the initial thin film below. These observations are direct evidence of an increase in viscosity upon further irradiation of the thin films of the catechol reaction mixture.

The kinetics simulations for thin films after irradiation, capturing the features of the experimental trends (see Figure 3b), provide further support for an increase in viscosity. While the diffusion coefficient of the reactive organic components is still unconstrained, within the wide range of 10^–20^ to 10^–10^ cm^2^ s^–1^, the diffusion coefficient of ozone in the thin film is significantly lower than before irradiation. At 0% RH, this diffusion coefficient is 3.5 × 10^–10^ cm^2^ s^–1^, compared to 4.5 × 10^–8^ cm^2^ s^–1^ before irradiation, as described above; at 50% RH, the value is 9.0 × 10^–8^ cm^2^ s^–1^, compared to 4.5 × 10^–7^ cm^2^ s^–1^. The effect of irradiation is most evident at 0% RH. A potential mechanism to increase viscosity upon irradiation is oligomerization, which has been reported for SOA from gas-phase oxidation of terpenes as well as primary BrC in thin films,^43,45^ through Norrish reactions,^73–75^ so we decided to also explore the effect of decreasing the initial number concentration of molecules that are reactive with ozone. To consider an extreme case, we reduced the initial number concentration after irradiation by a factor of 10 (see Table S4 and Figure S11). At 0% RH, the diffusion coefficient of ozone was not sensitive to this change in initial number concentration, remaining 3.5 × 10^–10^ cm^2^ s^–1^, still supporting a significant increase in viscosity. At 50% RH, the diffusion coefficient of ozone increased with this change in number concentration by about a factor of 5, from 9.0 × 10^–8^ to 5.0 × 10^–7^ cm^2^ s^–1^, roughly the same as the corresponding value before irradiation. If the light-driven change in viscosity occurs through oligomerization, then it is unlikely to so substantially reduce the number of ozone-reactive sites (i.e., unsaturated carbon–carbon bonds) through carbonyl photochemistry, so we conclude that the diffusion coefficient of ozone at 50% RH, like that at 0% RH, is reduced by irradiation.

Together, the agreement between the experimental and simulated time series of uptake coefficient and the qualitative observations of viscosity indicate that irradiation significantly increased the viscosity of the mixture of catechol and its reaction products. Previously, increased viscosity upon irradiation has been observed for SOA from the gas-phase oxidation of monoterpenes (i.e., α-pinene and limonene) as well as BBOA from the thermal degradation of wood (i.e., eastern red cedar).^43,45^ This increased viscosity has implications for the multiphase processing of these materials in the atmosphere, as explored in more detail below. The results here also show that the products of irradiation, potentially oligomers from Norrish reactions,^43^ remain sufficiently hygroscopic to absorb water and be plasticized as RH increases.^62^ At water saturation, both thin films may approach the viscosity of liquid water at the temperature used here, i.e., 293 K, as shown by Gregson et al.^53^

## Atmospheric Implications

Our results have implications for the processing and fate of phenolic species and their oxidation products in the atmosphere. To explore these implications, we implemented the physical and chemical parameters derived from the multilayer kinetics simulations of the experimental flow-tube results for a 300 nm-diameter particle at 293 K, exposed to ozone at a mixing ratio of 35 ppb. The same values of temperature and ozone mixing ratio were used previously to probe the multiphase processing of water-soluble and water-insoluble BBOA components, based on an earlier set of coated-wall flow-tube experiments.^44^ The resulting time series in Figure 4 show the concentration of reactive component in the aerosol particles relative to their initial concentration. In the model, every molecule of ozone lost to reactive uptake consumes one molecule of the reactive component. Note that the focus here is on constituents that react with ozone. Consequently, these time series do not represent the loss of light absorption, since reactions of phenolic species and their derivatives with oxidants may lead to products that also absorb light.^38,39,58^ Other aging mechanisms, like OH- and NO_3_-initiated oxidation, are not represented here, but they could also be important.^76–79^ Furthermore, we note that the precursor concentration used here in the aqueous oxidation is much higher than in cloud droplets, which could favor oligomerization over functionalization.^35,36^ Many studies have focused on identifying monomer and oligomer products of aqueous OH-initiated oxidation of phenolic compounds.^32–36^ Polar, oxygenated groups, including carbonyl groups, have been shown to be present in both,^35,36^ so it is reasonable to expect our observations of plasticization with water uptake and vitrification with irradiation to be broadly transferable to other distributions of reaction products. Finally, we note that, since the diffusion coefficient of the reactive components is uncertain within a wide range, there are three curves for each type of conditioning of the thin films, differing in the BrC diffusion coefficient, which is set to one of the three values introduced above: 1 × 10^–10^, 1 × 10^–15^, or 1 × 10^–20^ cm^2^ s^–1^.

**Figure 4:**
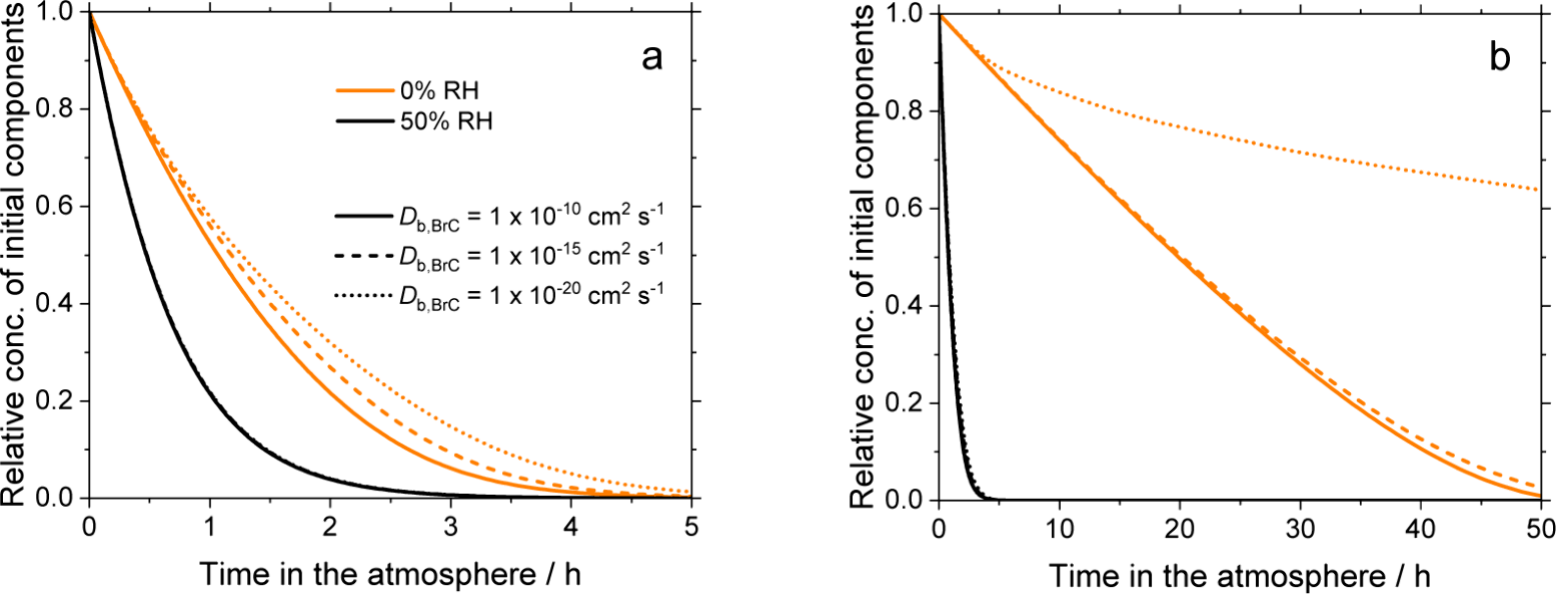
Relative concentrations of initial components of the reaction mixture in a 300 nm particle as a function of time in the atmosphere, at an ozone mixing ratio of 35 ppb and a temperature of 293 K, (a) without and (b) with further irradiation.

The estimated lifetimes of the reactive species are shortest for the materials and conditions with the highest reactive uptake of ozone in the flow-tube. The thin film without further irradiation exhibited at 50% RH the most reactive uptake, and the lifetime of the reactive components in this material is about 0.7 h, irrespective of the diffusion coefficient of the reactive organics (see Table S5). The next most significant reactive uptake occurred for the irradiated thin film at 50% RH. The estimated lifetime of the material with this conditioning is roughly 1 h, increasing slightly as the diffusion coefficient of the reactive organics decreased from 10^–10^ to 10^–20^ cm^2^ s^–1^. The next most significant uptake was observed at 0% RH for the thin film without further irradiation. Again, the impact of the diffusion coefficient is small, with the lifetime of this material at 0% RH increasing from 1.5 h at 10^–10^ cm^2^ s^–1^ to 1.8 h at 10^–20^ cm^2^ s^–1^. In Figure 4, all these conditions nearly overlap when compared to the time series of irradiated thin films at 0% RH, for which the shortest lifetime, at 10^–10^ cm^2^ s^–1^, is 26 h. Decreasing the diffusion coefficient by five orders of magnitude has little effect on the lifetime, still about 26 h. However, when the diffusion coefficient decreased another five orders of magnitude, the lifetime increased significantly, reaching about 170 h (i.e., about a week) in the atmosphere. Under these conditions, the diffusion of ozone from the surface into the bulk layers of the particle and of unreacted constituents from the bulk to the surface is restricted, and the initial constituents of the particles are long-lived. Since moderate RH values predominate in the ambient atmosphere near the surface,^41,42^ phenolic species and their product mixtures are likely to evolve in the particle phase due to multiphase processing, including the multiphase ozone oxidation studied here. As particles travel vertically in the atmosphere to regions of lower temperature, the physical and chemical properties could change accordingly,^56,63,80^ so in the future, lower temperatures could be explored to complete the perspective contributed here.

## Supplementary Material



## Notes

Sample preparation; absorbance, reactive uptake, and microscopy results; experimental parameters, best-fit diffusion coefficients, and lifetimes in 300 nm diameter particles (PDF)
