# Supplementary material for: Multiphase Ozone Oxidation of Catechol and Its Products after OH- and Light-Driven Processing
Source: ACS Earth Space Chem. 2025 Nov 7;9(11):2715–25. doi: 10.1021/acsearthspacechem.5c00230 (PMC12641540; doi:10.1021/acsearthspacechem.5c00230)
Supplement: Supplementary file 1 [file sp5c00230_si_001.pdf]

Supporting Information

Multiphase Ozone Oxidation of Catechol and Its Products after OH- and Light-Driven Processing

Sithumi M. Liyanage,[a] Meredith Schervish,[b] Habeeb H. Al-Mashala,[a] Katrina L. Betz,[a] Akansha Sharma,[a] Manabu Shiraiwa,[b] and Elijah G. Schnitzler*[a]

[a] *Department of Chemistry, Oklahoma State University, Stillwater, OK 74078, USA*

[b] *Department of Chemistry, University of California Irvine, Irvine, CA 92697, USA*

E-mail: elijah.schnitzler@okstate.edu.

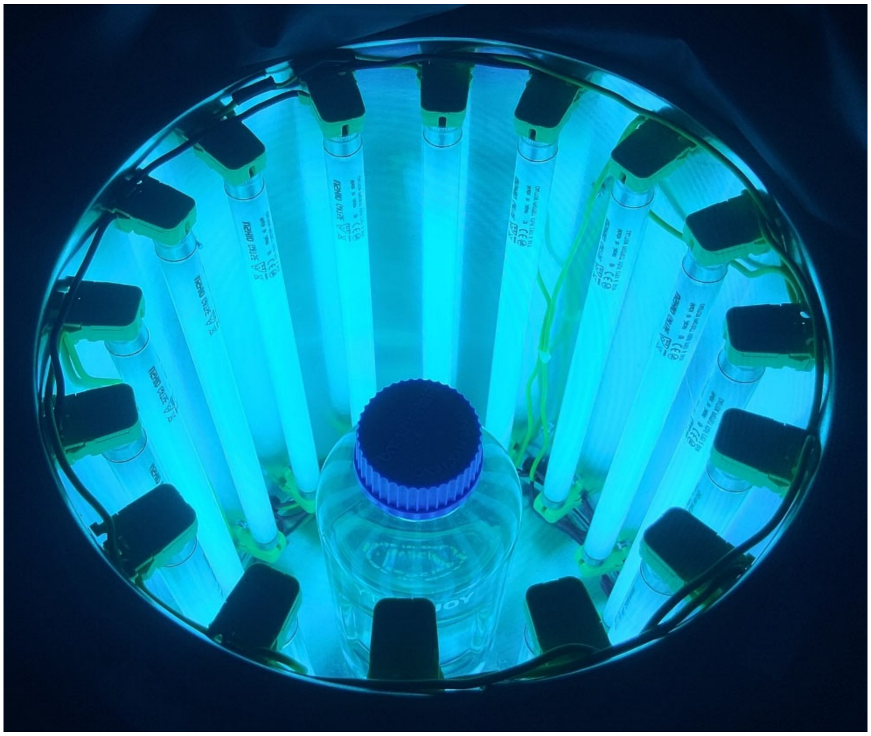

Figure S1. Photograph of custom-built photoreactor irradiating a glass reagent bottle.

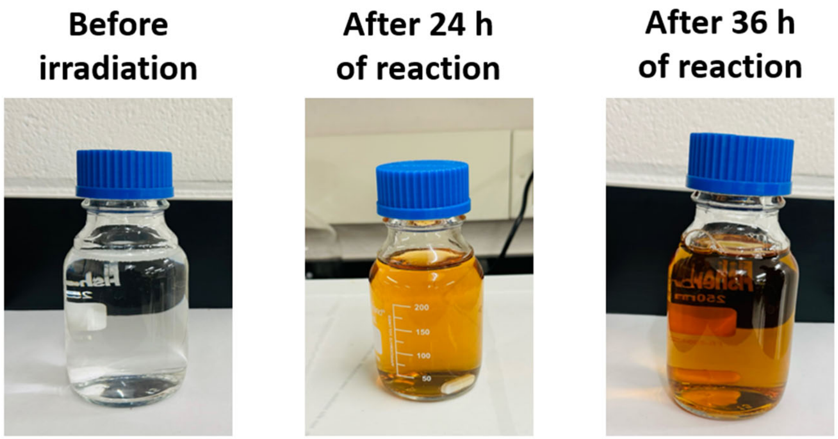

Figure S2. Photographs of the solution before and after photo-oxidation for a representative experiment in the photoreactor.

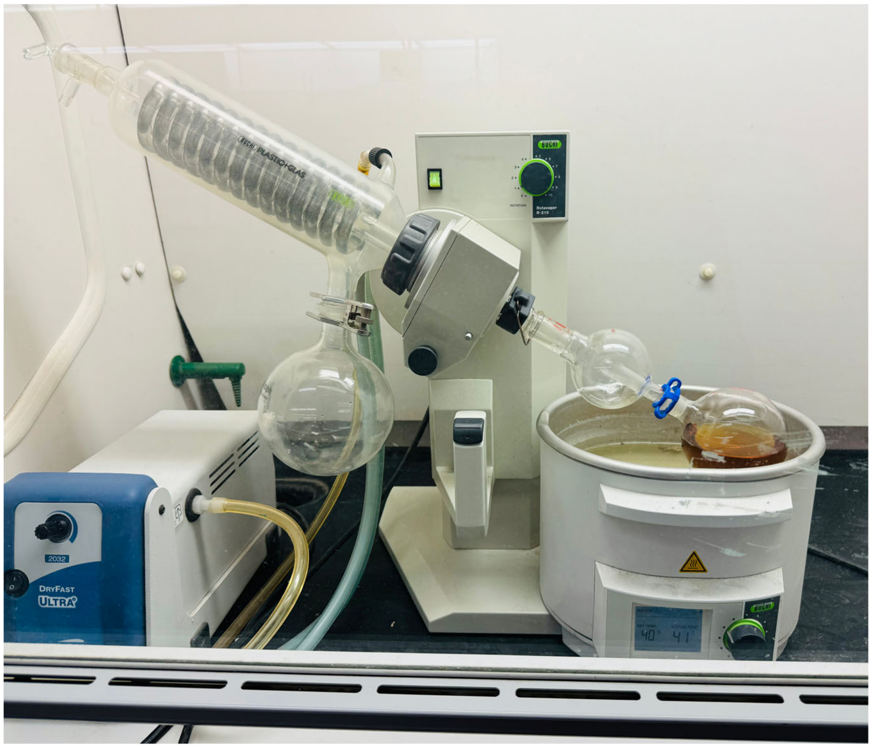

Figure S3. Photograph of rotary evaporator with the reaction mixture in the attached round-bottom flask.

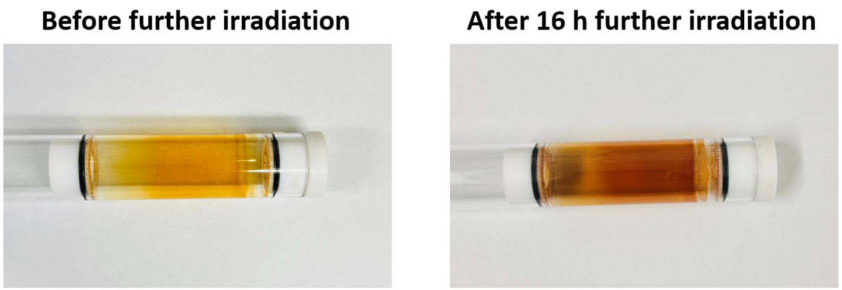

Figure S4. Representative thin film, prepared from the reaction mixture, before and after 16-h further irradiation as a thin film.

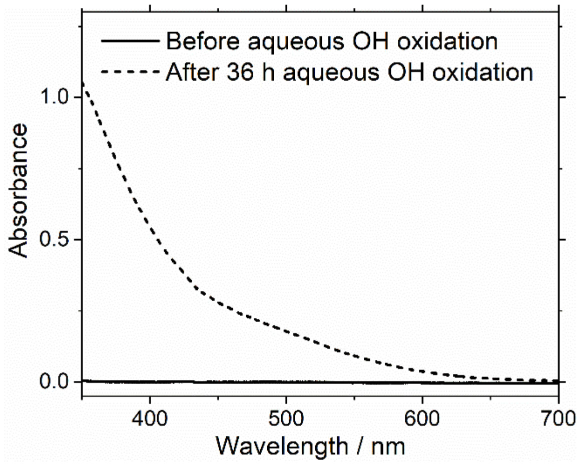

Figure S5. Absorbance of reaction mixture before and after aqueous OH-initiated oxidation in the photo-reactor.

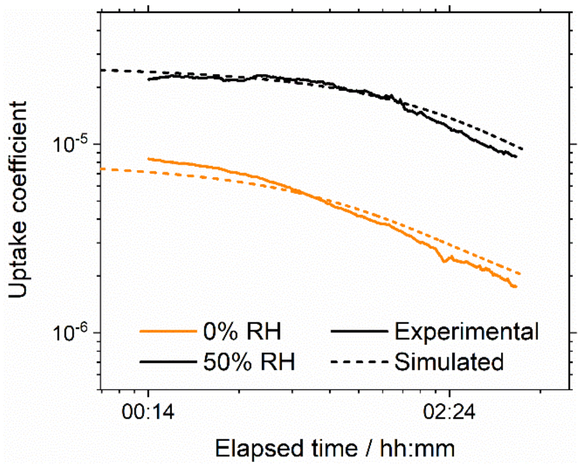

Figure S6. Log-log plot of uptake coefficients as a function of exposure time in the flow tube without further irradiation of the thin films. Each experimental time series is the average of triplicate experiments. The variance between triplicates is omitted here for clarity of presentation, but it is shown in terms of one standard deviation in Figure S7.

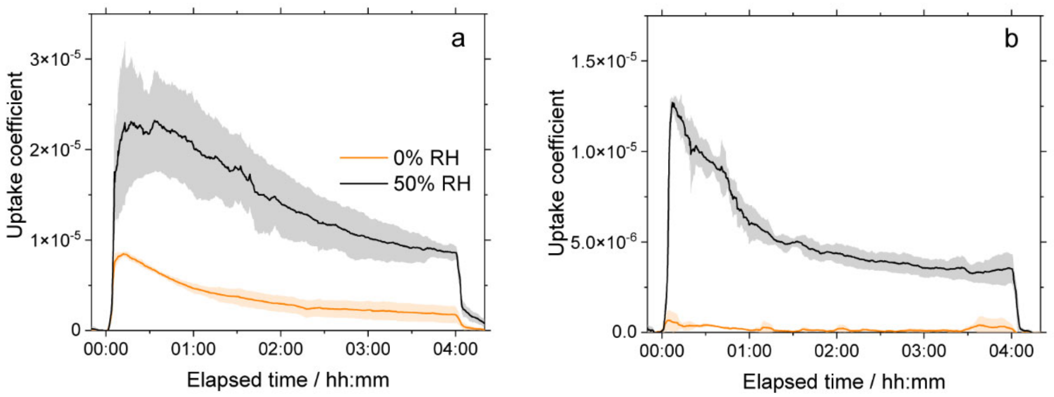

Figure S7. Experimental time series of uptake coefficient, depicting the average and variance of triplicate experiments (a) without and (b) with further irradiation of the thin films. The variance is shown in terms of one standard deviation.

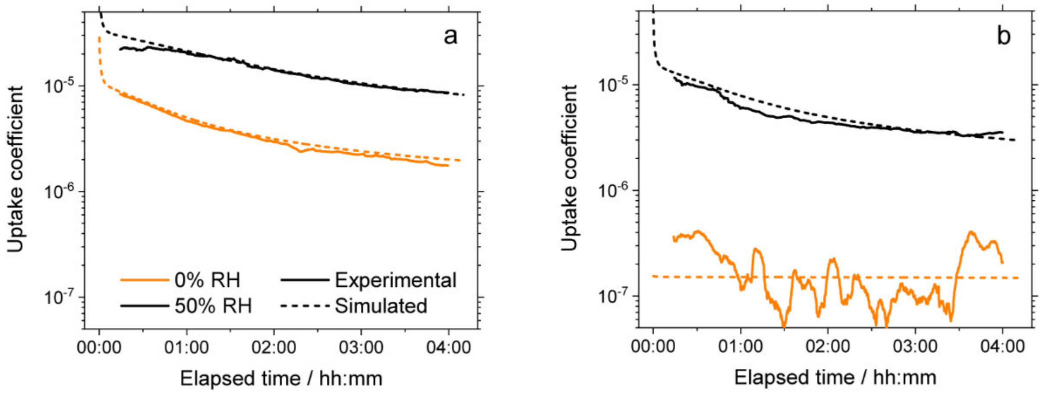

Figure S8. Uptake coefficients as a function of exposure time in the flow tube (a) without and (b) with further irradiation of the thin films, using parameters in Table S3, i.e., increasing the Henry's law constant of ozone by a factor of two. Each experimental time series is the average of triplicate experiments. The variance between triplicates is omitted here for clarity of presentation, but it is shown in terms of one standard deviation in Figure S7.

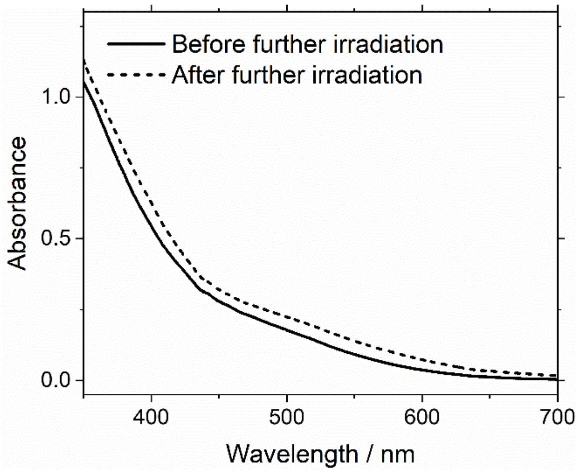

Figure S9. Absorbance of the reaction mixture before and after further irradiation of a thin film, measured at the same mass concentration in methanol.

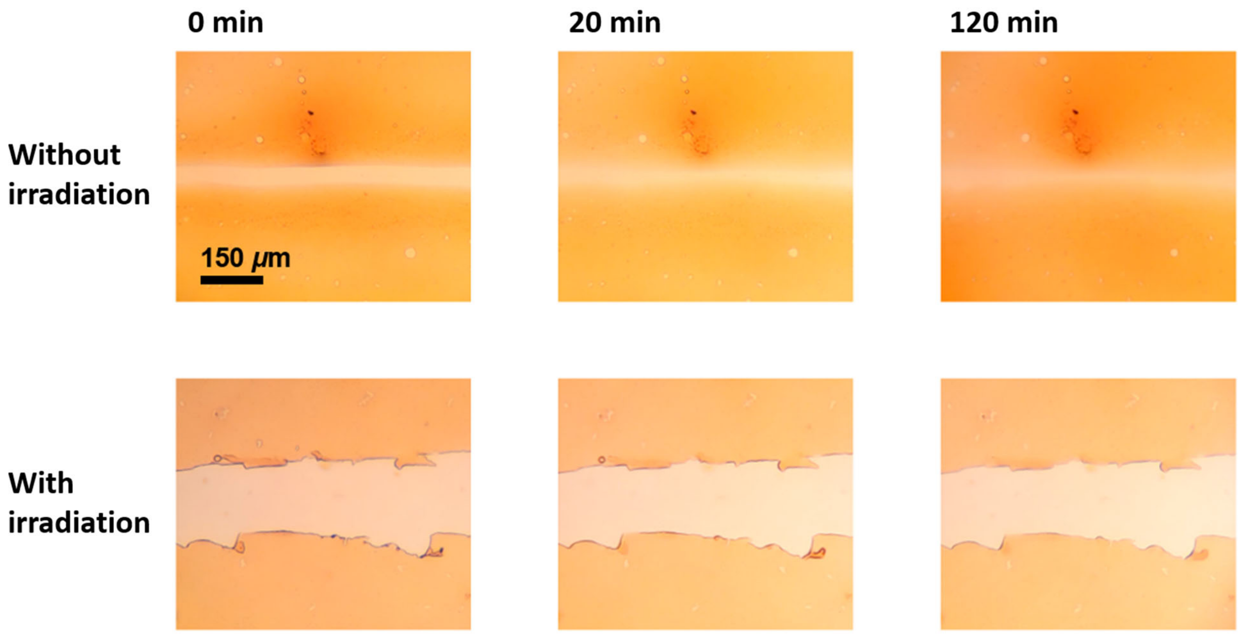

Figure S10. Photographs of flat thin films during microscopy experiments for samples without and with further irradiation, 0, 20, and 120 min after scraping.

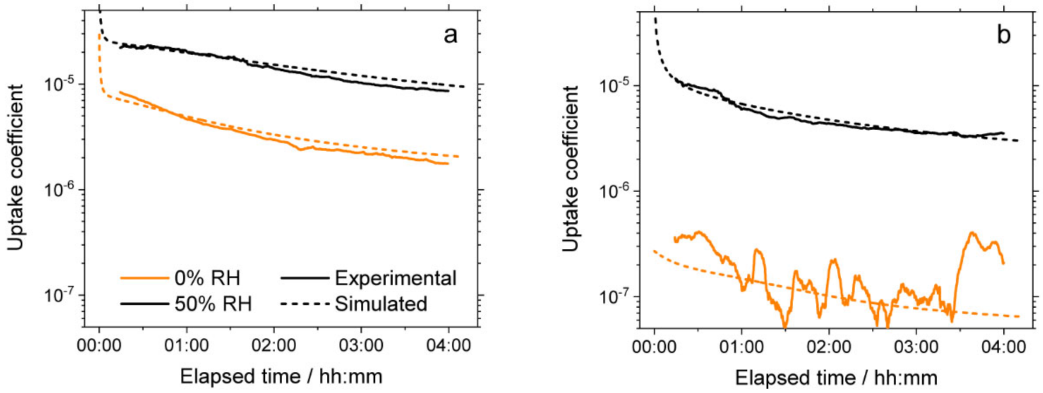

Figure S11. Uptake coefficients as a function of exposure time in the flow tube (a) without and (b) with further irradiation of the thin films, using parameters in Table S4, i.e., decreasing $[BrC]_{0,irr}$ by a factor of 10. Each experimental time series is the average of triplicate experiments. The variance between triplicates is omitted here for clarity of presentation, but it is shown in terms of one standard deviation in Figure S7.

Table S1. Parameters for the coated-wall flow-tube setup.

Parameter (symbol)	Value	Formula	Units
Temperature (T)	296.5		K
Pressure (P)	1		atm
Flow tube diameter (D_{tube})	2.45		cm
Flow tube cross-sectional area (A)	4.71	$A = \pi r^2$	cm^2
Volumetric flow rate (F)	0.30		L min^{-1}
Linear velocity (v)	1.06	$v = F/A$	cm s^{-1}
Length of coated tube (L)	5		cm
Residence time (t)	4.71	$t = L/v$	s
Reynolds number (Re)	17.3	$\mathrm{Re} = (\rho * D_{tube} * v)/\eta$	
Length to laminar flow (l)	1.48	$l = 0.035 * \mathrm{Re} * D_{tube}$	cm
Mean molecular velocity (ω)	362	$\omega = \mathrm{sqrt}(8RT/(\pi M))$	m s^{-1}
Ozone diffusion coefficient (D)	0.13		cm^2 s^{-1}
Mean free path (λ)	1.08E-05	$\lambda = 3D/\omega$	cm
Density of air (ρ)	1.2		kg m^{-3}
Viscosity of air (η)	1.80E-05		Pa s
Knudsen number (Kn)	8.81E-06	$\mathrm{Kn} = 2\lambda/D_{tube}$	
Dimensionless axial distance ($z*$)	0.204	$z* = z(\pi D/2F)$	
Sherwood number (N_{Shw})	4.10	$3.6568 + A/(z*+ B)$	

Table S2. Best-fit diffusion coefficients from the KM-GAP simulations with the initial concentration of reactive components, $[BrC]_0$, set to 3 x 10^{16} cm^{-3}; the second-order rate constant, k_{BR}, set to 5 x 10^{-17} cm^3 s^{-1}; and the Henry's law constant of ozone, HLC_{O3}, set to 2.4 x 10^{-4} mol cm^{-3} atm^{-1}.

**Material, condition**	$\boldsymbol{D_{b,O3}}$ **(cm^2 s^{-1})**	$\boldsymbol{D_{b,BrC}}$ **(cm^2 s^{-1})**
Not further irradiated, 0% RH	4.5 x 10^{-8}	1 x 10^{-20} – 1 x 10^{-10}
Not further irradiated, 50% RH	4.5 x 10^{-7}	1 x 10^{-20} – 1 x 10^{-10}
Further irradiated, 0% RH	3.5 x 10^{-10}	1 x 10^{-20} – 1 x 10^{-10}
Further irradiated, 50% RH	9.0 x 10^{-8}	1 x 10^{-20} – 1 x 10^{-10}

Table S3. Best-fit diffusion coefficients from the KM-GAP simulations with the initial concentration of reactive components, $[BrC]_0$, set to 3 x 10^{16} cm^{-3}; the second-order rate constant, k_{BR}, set to 5 x 10^{-17} cm^3 s^{-1}; and the Henry's law constant of ozone, HLC_{O3}, set to 4.8 x 10^{-4} mol cm^{-3} atm^{-1}. Here, HLC_{O3} is twice that in Table S2 while the other parameters are the same.

**Material, condition**	$D_{b,O3}$ **(cm^2 s^{-1})**	$D_{b,BrC}$ **(cm^2 s^{-1})**
Not further irradiated, 0% RH	2.0 x 10^{-8}	1 x 10^{-20} – 1 x 10^{-10}
Not further irradiated, 50% RH	1.8 x 10^{-7}	1 x 10^{-20} – 1 x 10^{-10}
Further irradiated, 0% RH	1.0 x 10^{-10}	1 x 10^{-20} – 1 x 10^{-10}
Further irradiated, 50% RH	4.0 x 10^{-8}	1 x 10^{-20} – 1 x 10^{-10}

Table S4. Best-fit diffusion coefficients from the KM-GAP simulations with the initial concentration of reactive components in the material without further irradiation, $[BrC]_{0,non\text{-}irr}$, set to 3 x 10^{16} cm^{-3}; the initial concentration of reactive components in the irradiated material, $[BrC]_{0,irr}$, set to 3 x 10^{15} cm^{-3}; the second-order rate constant, k_{BR}, set to 5 x 10^{-17} cm^3 s^{-1}; and the Henry's law constant of ozone, HLC_{O3}, set to 2.4 x 10^{-4} mol cm^{-3} atm^{-1}. Here, different values of $[BrC]_0$ are used for the materials without and with further irradiation; the value of $[BrC]_{0,irr}$ is 10 times lower than the value of $[BrC]_0$ in Table S2 to test the effects of a loss of initial reactive components, which would be attributed to oligomerization, while all other parameters are the same.

**Material, condition**	$D_{b,O3}$ **(cm^2 s^{-1})**	$D_{b,BrC}$ **(cm^2 s^{-1})**
Not further irradiated, 0% RH	4.5 x 10^{-8}	1 x 10^{-20} – 1 x 10^{-10}
Not further irradiated, 50% RH	4.5 x 10^{-7}	1 x 10^{-20} – 1 x 10^{-10}
Further irradiated, 0% RH	3.5 x 10^{-10}	1 x 10^{-20} – 1 x 10^{-10}
Further irradiated, 50% RH	5.0 x 10^{-7}	1 x 10^{-20} – 1 x 10^{-10}

Table S5. Lifetimes of initial components of the reaction mixtures in 300-nm diameter particles, T_{BrC}, for different values of the bulk diffusion coefficient of the mixture, $D_{\mathrm{b,BrC}}$, and different materials and conditions.

$D_{\mathrm{b,BrC}}$ ($cm^2\ s^{-1}$)	Material, condition	T_{BrC} (h)
1 x 10^{-10}	Not further irradiated, 0% RH	1.46
	Not further irradiated, 50% RH	0.68
	Further irradiated, 0% RH	26.0
	Further irradiated, 50% RH	1.07
1 x 10^{-15}	Not further irradiated, 0% RH	1.62
	Not further irradiated, 50% RH	0.68
	Further irradiated, 0% RH	26.3
	Further irradiated, 50% RH	1.14
1 x 10^{-20}	Not further irradiated, 0% RH	1.79
	Not further irradiated, 50% RH	0.68
	Further irradiated, 0% RH	167
	Further irradiated, 50% RH	1.18
